# Extracellular microvesicles/exosomes: discovery, disbelief, acceptance, and the future?

**DOI:** 10.1038/s41375-020-01041-z

**Published:** 2020-09-14

**Authors:** Mariusz Z. Ratajczak, Janina Ratajczak

**Affiliations:** 1 Stem Cell Institute, James Graham Brown Cancer Center, University of Louisville, Louisville, KY USA; 2 Department of Regenerative Medicine, Center for Preclinical Research and Technology, Medical University of Warsaw, Warsaw, Poland

**Keywords:** Developmental biology, Stem cells

## Abstract

There are concepts in science that need time to overcome initial disbelief before finally arriving at the moment when they are embraced by the research community. One of these concepts is the biological meaning of the small, spheroidal vesicles released from cells, which are described in the literature as microparticles, microvesicles, or exosomes. In the beginning, this research was difficult, as it was hard to distinguish these small vesicles from cell debris or apoptotic bodies. However, they may represent the first language of cell–cell communication, which existed before a more specific intercellular cross-talk between ligands and receptors emerged during evolution. In this review article, we will use the term “extracellular microvesicles” (ExMVs) to refer to these small spheroidal blebs of different sizes surrounded by a lipid layer of membrane. We have accepted an invitation from the Editor-in-Chief to write this review in observance of the 20th anniversary of the 2001 ASH Meeting when our team demonstrated that, by horizontal transfer of several bioactive molecules, including mRNA species and proteins, ExMVs harvested from embryonic stem cells could modify hematopoietic stem/progenitor cells and expand them ex vivo. Interestingly, the result that moved ExMV research forward was published first in 2005 in *Leukemia*, having been previously rejected by other major scientific journals out of simple disbelief. Therefore, the best judge of a new concept is the passage of time, although the speed of its adoption is aided by perseverance and confidence in one’s own data. In this perspective article, we will provide a brief update on the current status of, hopes for, and likely future of ExMV research as well as therapeutic and diagnostic applications, with a special emphasis on hematopoiesis.

## Introduction

Extracellular microvesicles (ExMVs), as we understand them today, are important players in cell–cell communication, tissue homeostasis, cell differentiation, as well as organ development and remodeling [1–5]. From an historical point of view, these small, spheroidal membrane blebs were first identified as being released from maturing reticulocytes and peripheral blood platelets [6, 7]. In the case of reticulocytes, the formation of ExMVs has been proposed as a mechanism to shed excess cell membrane and unnecessary surface receptors (e.g., transferrin receptors) during the maturation of these cells to erythrocytes, a process that requires a shrinkage of cell size [8]. In the case of platelets, platelet-derived ExMVs were identified as promoting procoagulant activity of blood by providing membrane platforms for the assembly of components of the coagulation protease cascade [9]. This activity is due to expression on the platelet surface of phosphatidylserine and tissue factor. However, over time, other biological effects have been assigned to these small membrane-derived fragments. They may, for example, transfer certain receptors from mentioned above reticulocytes and platelets and incorporate them into the plasma membranes of target cells, in this way changing the target cell phenotype [10–12] and even co-stimulating the proliferation of hematopoietic cells [13]. At about the same time, ExMVs were also reported to be shed from lymphocytes as supramolecular particles in association with membrane lipids or as antigen-presenting vesicles [14]. This field began to stabilize, and the presence of ExMVs could no longer be ignored. The belief that they are mere artifacts could not withstand the accumulating evidence of their involvement in biological processes.

It is interesting to recall how awareness of these intriguing vesicular structures developed. Everyone who has had experience analyzing established cell lines with cytometry can recall seeing small events in the lower left corner of the forward scatter (FSH) versus sides scatter (SSH) cytogram plot, which for a long time were considered by experienced cytometrists to be cell debris or apoptotic bodies. However, when evaluating the viability of investigated cell lines by FACS, there was no evidence that these cells had been damaged or had undergone apoptosis. Thus, an intriguing question emerged about whether the appearance of these spheroidal blebs could be a common phenomenon that occurs during cell activation and metabolism. Moreover, another question immediately followed: What is the overall biological significance of the release of ExMVs from cells?

As mentioned above, apoptotic bodies were the problem to move this research forward. At that time it was well known that dying and damaged cells release small cell fragments. Therefore, for many researchers, ExMVs were equivalent to apoptotic bodies. However, these latter vesicles are usually much larger and contain cell fragments due to disintegration of the cell by the proteolytic action of endogenous caspase 3, and they may be loaded with fragments of nuclei, nuclear DNA, and histones [15, 16]. A similar, albeit different, mechanism occurs in cells that are eliminated in the mechanism of pyroptosis [17]. However, pyroptosis, which also leads to cell blebbing, is preceded by cell swelling, lysis, and pore formation and is mediated by the proteolytic action of caspase 1. It is important to keep in mind these differences. However, there is the problem that some small apoptotic bodies or cell blebs from pyroptotic cells could be co-isolated with ExMVs. What is also important, apoptotic bodies similar to ExMVs may also interact with surrounding cells, and they usually provide a danger signal for innate immunity cells [15, 16].

The role of ExMVs is different, as they are released mostly from healthy, activated cells by proteolytic cleavage of the cytoskeleton or secreted from the endosomal cell membrane compartment [2–5]. They also play pleiotropic roles in cell–cell communication and affect several biological processes. They are present under steady-state conditions in all biological fluids investigated so far, including blood plasma, intercellular fluid, cerebrospinal fluid, urine, sperm, bile, synovial fluid, saliva, and breast milk. In pathologic situations the number of ExMVs increases in biological fluids, and they are also enriched in malignant effusions and ascites [18–20]. These pathology-associated ExMVs differ also in molecular composition.

Over time, biologically active ExMVs were found to be released from all types of cells in the adult organism, including normal and malignant hematopoietic [13, 18–20] and endothelial cells [21]. ExMVs can be internalized by target cells, mainly in the process of phagocytosis, and fuse with the target cell membranes, incorporating membrane fragments into the host cell membrane and delivering the ExMV cargo, composed of mRNA, miRNA, proteins, bioactive lipids, and signaling nucleotides, directly to the cytosol [21–26]. These two effects may change the phenotype of the target cell and modify its biological function. What will also be briefly reviewed in his review article is that the biological significance of ExMVs is based on their role as (i) signaling platforms, in which they stimulate cells with ligands embedded in their outer lipid layer, (ii) cell-surface phenotype “modifiers,” by transferring cell membrane receptors between cells, and (iii) cargo-delivery packets, by exchanging mRNA, miRNA, proteins, and some small organelles between cells, or, if engineered as vehicles, by transferring drugs, bioactive compounds, or genetic material to the target cells. Moreover, additional evidence has accumulated that ExMVs play a role in the spread of certain viruses and prions [2, 27, 28]. Currently under investigation is the possibility that ExMV molecular signature and content will be important tools in diagnostics as part of so-called liquid biopsies [20, 29].

Based on the foregoing, ExMVs have become an intensive subject of research. In the past 20 years our knowledge about ExMVs has expanded exponentially, and, at the time of this writing, if one searches PubMed by the term “microvesicles” or “exosomes,” one gets close to 7000 or 15,000 hits, respectively. By contrast, 20 years ago there were just a few citations, which were limited mostly to platelet-derived ExMVs. As of today, several seminal experimental papers and extensive reviews on this topic have been published. An international society exploring ExMVs has been created that publishes its own scientific journal (*Journal of Extracellular Vesicles*).

In this review paper, we will mainly focus on our scientific adventures working with ExMVs. This is why we chose the title “Extracellular microvesicles/exosomes: discovery, disbelief, acceptance.” We also added a question: “What will happen next?”

### The process of ExMV formation and their characterization

**Fig. 1:**
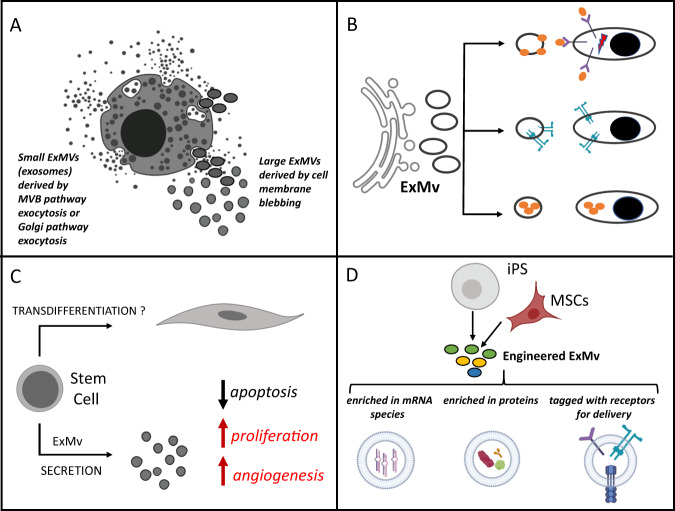
Cellular sources of ExMVs. **a** While larger ExMVs (100–1000 nm in diameter) are derived by cell-surface membrane blebbing, smaller ones, known as exosomes (50–150 nm), are derived by the multivesicular body (MVB) pathway or the Golgi apparatus pathway for exocytosis. **b** Biological effects of ExMVs. ExMVs may interact with receptors expressed on target cells by surface-expressed ligands (upper panel), transferring receptors to the target cells (middle panel), or transferring cargo containing mRNA, miRNA, proteins, or other biomolecules from one cell to another (lower panel). **c** The effect of mesenchymal stem cells employed as therapeutics in solid-organ regeneration. There is no convincing evidence that, after injection into damaged solid organs, mesenchymal stem cells, adipose stem cells, or hematopoietic stem cells can replace dying cells by trans-dedifferentiation. By contrast, all these cells may contribute to inhibiting apoptosis, increasing proliferation of surviving cells in a damaged organ, and promoting vascularization by acting as a source of paracrine factors, including those delivered by ExMVs. **d** Generation of therapeutic ExMVs. ExMVs may be produced from ex vivo-expanded MSCs in cell culture generators or from immortalized iPSCs. ExMV-producing cells could be engineered to produce ExMVs enriched in mRNA species and proteins that would provide pro-survival signals or be tagged with specific receptors for targeted tissue delivery. In the case of iPSC-derived ExMVs, it is important to also consider their potential unwanted tumorigenic potential.

Large ExMVs are released during cell-surface budding, and their sizes range from 100 to 1000 nm in diameter [1–5, 15, 16]. They are composed of an outer lipid bilayer and thus can be considered as “physiological liposomes,” in which a surface phospholipid bilayer surrounds inner content composed of mRNA, miRNA, noncoding RNAs, proteins (e.g., enzymes, signaling components, transcription factors), bioactive lipids (e.g., sphingosine-1-phosphate (S1P), prostaglandins, leukotrienes), signaling nucleotides, and metabolites (Fig. 1a). By contrast, smaller ExMVs, known as exosomes, are derived from the endosomal membrane compartment by budding of the endosomal membranes toward the interior of the endosome [2–5]. This creates endosomes that contain intraluminal vesicles, known as endosomal multivesicular bodies, which can release their content enriched in small exosomes after fusion with the plasma membrane into the extracellular space [15, 16]. Another source of small exosomes is the Golgi apparatus. Exosomes are smaller than cell-surface-derived ExMVs and are ~50–150 nm in diameter. What is important to keep in mind, ExMVs of different sizes are present in the extracellular space, and it is difficult to separate them. In studying the biological effects of the cell secretome in order to mimic the in vivo situation, the real biological impacts of these different-sized vesicles must therefore be evaluated together, even if there are differences in their size and molecular compositions.

It has been reported that larger ExMVs derived by cell membrane budding express CD40, selectins, integrins, and cytoskeletal proteins, and their membranes are highly enriched in cholesterol, phosphatidylserine, and diacylglycerol [15, 30, 31]. By contrast, small exosomes express specific markers, such as the tetraspanin family of proteins (e.g., CD63/CD9), thermal shock proteins (HSP70/90), and major histocompatibility class I antigens [15, 31].

There are different strategies currently available to characterize ExMVs, including (i) western blot, e.g., to detect tetraspanin components in the case of exosomes or certain cytoskeletal proteins expressed in cell membrane-derived ExMVs, (ii) nanoparticle tracking analysis, to calculate size distributions and numbers of ExMVs in suspension, and (iii) electron microscopy-based approaches and flow cytometry combined with antibodies against surface markers, for characterization of larger ExMVs. Other strategies for studying ExMV molecular signatures are based on molecular analysis of their cargo (mRNA species, protein, or bioactive lipid content) by employing “omics” technologies aimed at the universal detection of mRNA species (transcriptomics), proteins (proteomics), lipids (lipidomics), and metabolites (metabolomics).

### ExMVs as “signaling platforms”

There are several mechanisms by which single-celled organisms (e.g., bacteria or protozoa) and eukaryotic cells that are part of multicellular organisms may communicate with the surrounding environment and other cells in the body. As mentioned above, we may consider ExMVs as the first language with which cells started to communicate before more specific mechanisms mediated by peptides, bioactive lipids, extracellular nucleotides, and their specific receptors emerged during evolution. It has been initially speculated that spheroidal membrane fragments shed from hematopoietic cells may be critical in the differentiation and proliferation of these cells [32]. Recognized relatively early was that ExMVs may express on their surface certain molecules embedded in the lipid bilayer that, if presented to specific receptors on target cells, activate signaling cascades.

From early development on, ExMVs are involved in intercellular cross-talk. An example is argosomes, which are involved in tissue patterning during embryogenesis by creating morphogen gradients [33]. These ExMVs express the Wingless morphogen on their surface, and in developing *Drosophila* the imaginal disc epithelium serves as a vehicle for the spread of Wingless protein over large distances [33]. Evidence has accumulated that ExMVs also mediate embryo and maternal interactions at implantation of blastocyst and during pregnancy [34].

Therefore, it is not surprising that our team initially employed ExMVs isolated from both embryonic murine and human stem cells to test their biological effects in a model of ex vivo expansion of hematopoietic stem/progenitor cells (HSPCs) [23]. We found that the Wingless signaling ligand Wnt-3, expressed on the surface of these ExMVs, was involved in ex vivo expansion of murine and human HSPCs. Next, we employed platelet-derived ExMVs and discovered that they chemoattract human CD34^+^ cells as well as cells from established human hematopoietic cell lines [23]. Platelet-derived ExMVs also increased the adhesion, proliferation rate, and survival of these cells, and activated various intracellular signaling cascades in these cells, including p42/44 MAPK, the PI3K–AKT pathway, and STAT proteins [23]. The biological effects of these ExMVs when employed as signaling platforms were only partly reduced by heat inactivation or trypsin digest, indicating that, in addition to the protein components, lipid components were also responsible for their biological activity. Today it is well known that platelet-derived ExMVs express S1P, which was most likely responsible for several of the observed effects including hematopoietic [23, 35].

Based on these findings, this developmental mechanism is preserved in adult tissues, as seen in cell-to-cell short, and even remote, communication. During blebbing from cell-surface membranes or after originating from the endosomal compartment, ExMVs can “hijack” ligands embedded in their lipid bilayer that can then interact with receptors on target cells (Fig. 1b). These ligands could be membrane-expressed peptide ligands as well as bioactive lipids or extracellular signaling nucleotides associated with ExMVs. These signaling interactions may regulate tissue homeostasis, and ExMV cross-talk plays an important role in the interaction of HSPCs with the bone marrow (BM) microenvironment and hematopoietic niches [15, 31, 36, 37]. However, this interaction is a two-way street, since stem cell niche-derived ExMVs may regulate the development and differentiation of HSPCs, while, vice versa, HSPC-released ExMVs affect the physiological functions of the stem cell niche. To get the full picture, we have to look at the BM microenvironment as a space filled with ExMVs derived from different cell types present in this tissue, and this illustrates the complex nature of this cell–cell communication system. An analogous cross-talk mechanism between different cell types operates in other tissues as well. It is also involved in cross-talk between cells in pathological conditions, such as cancerogenesis, which will be discussed below. A pathological BM microenvironment is usually more enriched in ExMVs, and their compositions are different from those observed in steady-state conditions [18–20].

### ExMVs in receptor transfer between cells

During cell membrane blebbing, some of the cell-surface-expressed receptors and markers may be incorporated as mentioned above into the outer layer of ExMVs and transferred by membrane fusion to the target cell membrane. This process may change the phenotype and some of the biological properties of the recipient cells after transfer (Fig. 1b). As an example, almost 20 years ago we tried to isolate megakaryocytic progenitor cells from BM assuming that they would be CD34^+^ and positive for the megakaryocytic lineage-specific integrin alpha chain 2 beta (CD41). To our surprise, we discovered by FACS analysis that up to 60% of the CD34^+^ cells isolated after leukopheresis from mobilized peripheral blood were positive for CD41 antigen, and after in vitro testing the CD34^+^CD41^+^ phenotype did not correspond with enhanced CFU-Meg potential relative to CD34^+^CD41^−^ cells [12]. This somewhat surprising result was explained by the fact that CD34^+^ cells during leukopheresis became covered with CD41^+^ ExMVs derived from platelets activated in the plastic tubing during the leukopheresis procedure [12]. This was evidence that ExMVs may transfer receptors to cells and change their surface phenotype. In the next step, we employed this observation to “cover” murine BM-isolated SKL cells with platelet-derived ExMVs and demonstrated that transfer of CD41 receptor to these cells increased their interaction with BM endothelium and enhanced BM seeding efficiency of these cells after transplantation [12]. This strategy was successful in experimental murine models and now awaits potential testing in the clinic.

As we reported later, this phenomenon of receptor transfer by platelet-derived ExMVs could also be involved in increasing the metastasis of cancer cells [38]. Specifically, tumor cells may acquire expression of CD41 antigen from peripheral blood platelets activated by tumor cells expressing tissue factor, and in consequence this receptor is transferred to tumor cells by platelet-derived ExMVs. This process has been demonstrated in a model of metastasis using human lung cancer cell lines [38]. Similarly, as we have demonstrated, the CXCR4^+^ receptor can be transferred with ExMVs from platelets and megakaryocytes to other cells and thereby facilitates infection of CD4^+^ target cells by T-tropic HIV [27]. This phenomenon of receptor transfer by ExMVs was subsequently demonstrated by other investigators in various experimental situations. It is important to keep in mind that, during processing and purification of cell fractions enriched in HSPCs with a FACS sorter, some of the cell-surface antigens can be incorporated by ExMVs into sorted cells and make them false positives for lineage markers. As we have demonstrated, this situation can occur after lysis of red blood cells in hypotonic solution in which erythrocyte-derived blebs may transfer the erythrocyte marker glycophorin A (GPA-A) and phosphatidylserine to hematopoietic stem cells and mark them falsely as cells from the erythroid lineage or even as cells undergoing early apoptosis [10]. This problem has to be kept in mind when using hypotonic lysis during preparation of a cell suspension, for example, for FACS analysis.

Overall, this mechanism of receptor transfer between cells involving ExMVs has been confirmed in several excellent papers, and because of space constraints we cite only a few of them here [31, 39–43]. However, more results are needed to see whether some of the receptors transferred by ExMVs retain their full signaling properties.

### ExMVs as “cargo-delivery packets”

ExMVs and their cargo, which can be enriched in any of several bioactive mediators that we know of today, can, depending on their size, be internalized into cells by different mechanisms. These include phagocytosis, caveolin-, clathrin-, or lipid raft-mediated endocytosis, micropinocytosis, and, what is crucial for their biological effects, by direct membrane fusion (Fig. 1b). If they are not degraded by lysosomes, ExMVs release their bioactive cargo into the cytosol. This latter phenomenon is well documented and is the basis for explaining how cargo imported via ExMVs may change the biology of target cells.

We have believed that this could be an important mechanism, and in the abovementioned work we showed for the first time that embryonic stem cell-derived ExMVs may transfer several biologically relevant molecules (mRNA species and proteins) to HSPCs [23]. This occurs during ExMV-directed ex vivo expansion of human and murine HSPCs. We found that ESC-derived ExMVs enhanced cell survival, improved the expansion rate, and upregulated the expression of early pluripotent stem cell markers (Oct-4, Nanog, and Rex-1) and early hematopoietic stem cell markers (Scl, HoxB4, and GATA 2) in these cells [23]. Following this report, other very elegant studies confirmed in subsequent years the presence of horizontal transfer of mRNA species via ExMVs in models of glioblastoma [15], murine and human mast cells [25], lung cells [24], endothelial cells [21], and mesenchymal stem cells [44]. Specifically, glioblastoma-derived ExMVs were found to be enriched for mRNA, miRNA, and proangiopoietic proteins and, after horizontal transfer of these molecules, promoted angiogenesis in growing tumors and stimulated tumor cell proliferation [15].

### The role of ExMVs in stem cell therapies and tissue/organ regeneration

Tissue and organ regeneration after damage is one of the leading topics in contemporary medicine. Different types of stem cells of embryonic origin have been proposed, including adult cells genetically modified to attain a state of pluripotency, known as induced pluripotent stem cells (iPSCs), and stem/progenitor cells, such as those isolated from adult tissues as potential therapeutics to mend damaged organs [45]. However, despite the well-known fact that HSPCs have been successfully employed for almost half a century to treat hematological malignancies or certain inborn metabolic diseases, there is a problem with therapeutic application of these and other stem cells for regeneration of solid organs and nonhematopoietic tissues. Stem cells isolated from embryos and iPSCs have not fulfilled expected goals, and, moreover, they carry a risk of teratoma formation, and deep sequencing studies and chromosomal analyses have revealed several changes in these cells related to genomic instability [46]. Thus, it seems that, as for now, the only potential application for these established pluripotent cell lines derived from embryos or by genetic manipulation is as sources of paracrine factors, including as a source of ExMVs for therapy [45]. Here, however, more careful studies need to be performed to determine whether there is a risk that ExMVs derived from these cells may in some rare cases reprogram somatic cells in damaged organs to a state of malignancy. Taking into consideration the possibility that, during the production of therapeutic ExMVs from iPSCs, conditioned media in cell-expansion generators contains DNA fragments from cells that have undergone apoptosis, such a risk may exist. In fact, it has been demonstrated in rare cases that DNA from malignant cells can be transferred to normal cells [47].

At the same time, stem cells isolated from adult tissues, including mesenchymal stromal cells (MSCs), adipose tissue cells, and myoblasts, have so far yielded no solid evidence that these cells contribute to replacement of damaged tissue cells in humans. The only documented effects from the application of these cells in regenerative medicine are that, after delivery to damaged organs, in a paracrine manner they may inhibit apoptosis of cells, promote angiogenesis for a better blood supply, and in some cases stimulate cells that have survived in damaged tissues to proliferate in order to replenish dying tissue fragments (Fig. 1c). Interestingly, similar effects to those observed after the delivery of intact MSCs were observed after therapeutic delivery of MSCs-derived ExMVs [3, 44]. These observations became particularly important at the time when the concept of stem cell plasticity held sway, when some of the markers derived from cells employed as therapeutics were detected in cells in the damaged tissues. We believe that this phenotypic modification of target cells in damaged organs can be explained better by the transfer of cell-surface markers by ExMVs derived from cells employed as therapeutics rather than by the phenomenon of cell fusion [2, 23, 48].

Taking all this into account, it has been proposed that instead of applying intact cells for the purpose of regenerative medicine, it would be better to employ ExMVs harvested from these cells and expanded ex vivo as a potential source of therapeutic ExMVs [45]. To confirm this in a seminal paper it was demonstrated that MSC-derived ExMVs have the same therapeutic effect on kidney damage as intact MSCs [49]. This observation has been confirmed by other investigators and establishes a path to employing ExMVs as therapeutics. It has already been 10 years since we proposed that ExMVs could have an important role in regenerative medicine and that ExMV-producing cells (e.g., MSCs) could be engineered to overexpress antiapoptotic and proangiopoietic factors (growth factors, cytokines, surface molecules, mRNA, and miRNA) and could be harvested from large-scale in vitro cultures of these ExMV-producing cells for therapeutic applications (Fig. 1d) [45]. Such custom-engineered stem cells and “super MVs” could inhibit apoptosis of target cells, stimulate cells that have survived damage to proliferate, and promote neovascularization of damaged tissues, thereby serving as a new class of cell-derived therapeutics in regenerative medicine. As of today, ExMVs have been successfully applied instead of intact cells in several experimental settings in animals to regenerate injuries to kidney, myocardium, and the central nervous system and to treat liver fibrosis [44, 49–51]. These encouraging results await further solid confirmation in the clinical setting. This possibility is now being intensively investigated and is the subject of an initial clinical trial. Another question that needs to be better addressed is that, since producer cells also secrete several soluble factors in addition to ExMVs, should they be removed from the cell secretome or employed together with ExMVs? As mentioned above, some reports claim that ExMVs are not only equally efficient but even more efficient in the regeneration of damaged tissues as are the cells that are the source of these small membrane blebs.

Currently, which is important for hematologists, there are also attempts to employ ExMVs isolated from MSCs in experimental hematology to mitigate the postirradiation damage to hematopoiesis or to speed up hematopoietic recovery after hematopoietic transplantation in animal models [31, 52]. ExMVs have a beneficial effect on the regeneration of BM damaged by irradiation, as they reduce early ionization toxicity and apoptosis of cells in the BM microenvironment and provide protection to BM blood vessels. Because of their immunosuppressive properties, MSC-derived ExMVs are also being employed to mitigate GvHD in animal models [53] and this strategy is awaiting first clinical trials in humans [31]. For these applications, MSCs, as producers of ExMVs, could be isolated from BM adipose tissue or umbilical cord blood Wharton jelly [31, 53, 54].

### The role of ExMVs in leukemia and solid tumors

All the biological ExMV effects identified for normal cell–cell communication are also involved in malignant cell transformation and cancerogenesis. They play an unwanted role in progression or even potentially in the initiation of tumorogenesis, which needs to be more carefully investigated. Tumor cells are a rich source of ExMVs, which affect not only other malignant cells in expanding cancer but may also modify the surrounding tissue where the tumor is growing and decrease the immune response. On the other hand, ExMVs derived from normal cells may also modify tumor growth and metastasis, which demonstrates again the existence of a “two-way street” for their involvement in cell–cell communication.

The molecular signature of leukemic ExMVs varies, depending on the type of malignancy and the stage of the disease [18, 20, 55–58]. They also have different molecular profiles than ExMVs derived from normal hematopoietic cells. These differences are observed mainly in the expression of certain surface markers and in their inner cargo of miRNAs and proteins. There are several excellent papers showing the role of ExMVs in leukemia. Specifically, ExMVs may directly or indirectly affect normal HSPCs by modifying hematopoietic niches and other cells in the BM microenvironment or directly inhibiting their growth and differentiation [15, 31, 55–58]. On the other hand, in some cases cells may also be forced by the hematopoietic microenvironment to release ExMVs that increase chemoresistance, inhibit apoptosis, and increase the quiescence of malignant clones. As has been shown previously, this occurs by changing the repertoire of growth factors and cytokines that are more advantageous for the expansion of leukemic cells. Tumor-derived ExMVs may also stimulate angiogenesis and endothelial cells as sources of several growth and antiapoptotic factors that may play a role in leukemia progression [59, 60]. Another important factor promoting leukemia expansion is the role of ExMVs in inhibition of immune defenses, as demonstrated by their effects in promoting (i) apoptosis of cytotoxic T cells, (ii) cytotoxic effects against NK cells, and (iii) impaired differentiation of dendritic cells. Leukemia cell-derived ExMVs may also convert monocytes into “tumor-associated macrophages,” which release tumor-supporting growth factors [61]. An open question is relevant to the abovementioned application of iPSC-derived ExMVs (Fig. 1d): Do these small carriers of mRNA species and proteins derived from immortalized cells also have similar unwanted effects?

### Application of ExMVs to diagnostics and drug delivery

Since ExMVs often have a unique molecular signature that depends on the cell of origin, they could serve as a kind of business card with which it is sometimes possible to identify the parent cells. This has opened the door to exploring their application as diagnostic tools to identify pathologic changes in the body. Based on this approach, ExMVs have emerged as an important diagnostic tool in liquid biopsies as noninvasive, pain-free approach to diagnose, monitor, and to overcome the limitations of traditional tissue biopsies [31, 39]. This exciting area of research is still in its infancy, although the first promising reports have been published. There have been attempts to create databases, such as ExoCarta or Vesiclepedia 2019, for molecular cargos from different ExMVs. Thus, deciphering the molecular ExMVs components may potentially alter medical practice, as it is hoped that medical diagnostics will use this information for hematological disorders.

On the other hand, taking advantage of the fact that ExMVs may have advantages over synthetic liposomes or nanoparticles and may very well protect their inside cargo by a membrane bilayer, they could be harnessed for delivery of drugs or genes. The first trials have already been reported for ExMVs encapsulating antileukemia drugs, such as imatinib, paclitaxel, and doxorubicin, or the anti-inflammatory compound curcumin [62–65]. The advantage of ExMVs is that, because of their small size, they can penetrate the blood–brain barrier and, for example, target leukemic cells that have infiltrated the central nervous system [66].

Recently, it has been proposed to employ ExMVs derived from animal milk or exosome-like carriers from plants for drug delivery [67]. They could be delivered through the digestive tract, and they have proven to be stable, biocompatible, and resistant to digestive enzymes. These areas of ExMV application are rapidly expanding, and we expect new and intriguing publications on these topics.

### Problems and future directions

As with every new technology, there are still some problems in optimizing the isolation protocols for ExMVs, their characterization, and tracking after delivery in vivo, and their efficient, clinical-grade production protocols according with GMP requirements [68]. The methods employed so far for their isolation and purification are based on initial centrifugation of collected cell supernatants to remove cell debris, followed by ultracentrifugation of cell-free extracts on density gradients, size exclusion chromatography, filtration, precipitation, the use of magnetic or agarose beads, and combinatory approaches using these methods. Most of these strategies are still under development, in particular for clinical-grade purification of ExMVs. There have been attempts to identify the specific molecular signatures of ExMVs that are circulating in peripheral blood or are present in other biological fluids to track their origin from normal or malignant cells. Another important problem is the efficient targeted delivery of ExMVs administered for therapeutic purposes. Another problem is their relatively rapid clearance from peripheral blood, and thus there is a need to develop approaches to extend their life span so that they can fulfill their therapeutic goals. For in vivo applications one also has to consider off-target effects and insufficient internalization of the ExMV cargo into target cells.

Another important question is whether, in the case of unwanted effects of ExMVs as seen in certain pathologies, it is feasible to remove them from the PB to attenuate their biological effects. Based on the role they may play in tumor progression, immunosuppression of activated CD8^+^ T cells, or in promoting drug resistance between cancer cells by horizontal transfer of miRNAs regulating resistance to chemotherapeutics, it is reasonable to attempt to inhibit their release from tumor cells or even eliminate them from peripheral blood. There are potential strategies, including plasmapheresis and filtration, to decrease the burden of circulating ExMVs; to employ experimental treatments that inhibit their formation, such as application of dimethyl amiloride; or to inhibit their fusion with target cells after blockade of ExMV-expressed phosphatidylserine with diannexin. The benefit of these ExMV-inhibiting strategies, however, needs further clinical verification that they will not interfere with the presence of or the physiological role of normal ExMVs. Moreover, since platelet-derived ExMVs may, after the transfer of several endothelium-targeted adhesion receptors, render tumor cells more metastatic, one of the important precautions is not to employ outdated platelets, which are enriched in ExMVs, in low-platelet-count cancer patients. This should be easily achieved by employing fresh platelet units for substitution therapies [38]. Similar concern applies to stored erythrocytes that undergo structural and biochemical alterations and release procoagulant ExMVs [69].

## Conclusions

Evidence has accumulated that ExMVs participate in almost all biological processes in the body, and, remarkably, this was unappreciated until recently. Cross-talk between cells mediated by ExMVs is involved in maintaining tissue homeostasis during tissue and organ regeneration, angiogenesis, and in pathologies such as cancerogenesis, complications of chronic inflammation, and atherosclerosis. Further research is needed to better decipher the molecular signature of mRNA species (mRNA, miRNA, and long noncoding RNA), proteins, bioactive lipids, and signaling nucleotides in ExMVs isolated from normal human individuals of different sexes and ages, as well as in patients presenting various health problems. What is even more important, this knowledge has to be better translated at the mechanistic level to explain the biological effects of ExMVs, as this may shed more light on the pathogenesis of various diseases. There are also other problems to be solved, including a persistent lack of well-established, rapid, and standardized methods for isolating ExMVs; measuring their number; and purifying them efficient from biological fluids. We need also to identify better stimuli that promote release of ExMVs from stressed and activated cells. Moreover, how important in their generation in addition to other stimuli is activation of innate immunity and purinergic signaling? [70]. Thinking about their therapeutic application in the clinic, we also have to consider potential “off-target” side effects of such therapy, including for example the risk of hypercoagulation or, in the case of ExMVs derived from iPSCs, the possibility of transfer of cargo that may potentially promote neoplastic transformation of target cells. Nevertheless, there is no doubt that, in answering the question from our title “what will happen next?,” we will continue to see rapid progress, particularly with new therapeutic and diagnostic applications of ExMVs. Let us stay alert to new developments!
